# The association of hamstring tightness with lumbar lordosis and trunk flexibility in healthy individuals: gender analysis

**DOI:** 10.3389/fbioe.2023.1225973

**Published:** 2023-09-14

**Authors:** Nesma M. Allam, Hasnaa Ali Ebrahim, Ateya Megahed Ibrahim, Nora Helmi Elneblawi, Mohamed El-Sherbiny, Khaled Zaki Fouda

**Affiliations:** ^1^ Physical Therapy and Health Rehabilitation Department, College of Applied Medical Science, Jouf University, Sakaka, Saudi Arabia; ^2^ Physical Therapy Department for Surgery, Faculty of Physical Therapy, Cairo University, Giza, Egypt; ^3^ Department of Basic Medical Sciences, College of Medicine, Princess Nourah bint Abdulrahman University, Riyadh, Saudi Arabia; ^4^ Department of Nursing, College of Applied Medical Sciences, Prince Sattam bin Abdulaziz University, Al-Kharj, Saudi Arabia; ^5^ Department of Family and Community Health Nursing, Faculty of Nursing, Port Said University, Port Said, Egypt; ^6^ Department of Medical-Surgical Nursing, College of Nursing, Taibah University, Madinah, Saudi Arabia; ^7^ Department of Basic Medical Sciences, College of Medicine, Almaarefa University, Riyadh, Saudi Arabia; ^8^ Department of Basic Science for Physical Therapy, Faculty of Physical Therapy, Cairo University, Giza, Egypt

**Keywords:** hamstring tightness, lumbar lordosis, trunk flexibility, flexible ruler, healthy individuals

## Abstract

**Objectives:** The purpose of this study was to investigate if there is a relation between hamstring tightness and lumbar lordosis as well as trunk flexibility based on gender differences and to analyze the differences in hamstring tightness, lumber lordosis and trunk flexibility in healthy adults.

**Methods:** One hundred young healthy adults were recruited and distributed into 2 equal groups according to gender: group A (female group) and group B (male group). Hamstring tightness (HT) was measured by Active Knee Extension (AKE) test and Straight Leg Raise (SLR) test, the angle of lumbar lordosis was measured with a flexible ruler from standing position and trunk flexion flexibility (TFF) was measured by Fingertip-to-Floor Test.

**Results:** There was a significant correlation between TFF and both measures of HT (SLR, *p* = 0.001; AKE, *p* = 0.001) in females. While, there was a non-significant correlation in males (SLR, *p* = 0.900; AKE, *p* = 0.717). Moreover, there was a non-significant correlation between lumbar lordosis and HT measures in both groups as (*p* > 0.05). Furthermore, there were significant differences between males and females in hamstring flexibility, TFF and lumbar lordosis as (*p* < 0.05).

**Conclusion:** Gender differences in the relationship between hamstring tightness and trunk flexion flexibility are significant. However, there was no significant difference between males and females in the relationship between hamstring tightness and lumbar lordosis.

## Introduction

Some of the most prevalent musculoskeletal problems among modern students are functional disorders of posture (Gorelov et al., 2013; Dud and ko, 2015). Sedentary lifestyle contributes to decreased hamstring flexibility due to adaptive shortening of the musculature, tendons, and fascia, which are maintained for an extended period of time at some angle of contraction, such as in prolonged sitting position (Liu et al., 2012).

Hamstring tightness is commonly defined as a decrease in range of motion with a feeling of restriction in the posterior thigh. It can occur as a result of a number of factors, including muscular injury, genetic predisposition, and compensatory shortening as a result of chronic deformities (Spinoso et al., 2022). Hamstring tightness results in slight knee flexion throughout activities and inputs relatively high quadriceps forces to counteract the passive resistance of the hamstring. This may increase the reaction forces at the patellofemoral joint and cause knee joint pain that impairs gait (White et al., 2009).

Hamstring flexibility is regarded as the most important muscle influencing pelvic position. It helps to stabilize the pelvis in the sagittal plane by attempting to control anterior pelvic tilt and trunk forward bending during dynamic posture. Tightness of the hamstring muscles during spine flexion can limit anterior pelvic tilt, causing lumbar muscle and ligamentous tension to worsen, resulting in much higher compressive pressures on the lumbar spine because bending forward is among the most common movements in daily activities (Norris and Matthews, 2006; Lopez-Minarro and Alacid, 2010; Cejudo et al., 2021).

The active knee extension (AKE) and straight leg raise (SLR) tests are frequently regarded as the gold standards for assessing hamstring flexibility, as they can indirectly measure hamstring muscle length. The AKE test is used to evaluate hamstring muscle length and active knee extension range in hip flexion position. Knee extension deficit is a measure of hamstring flexibility (Hansberger et al., 2019).

The gold standard technique for measuring lumbar lordosis is X-ray radiography, but it is costly and dangerous due to exposure to radiation. In clinical and larger populations, a flexible ruler is an alternative non-radiographic, economical, and non-invasive method to assess the degree of curvature of the spine in the sagittal plane, such as kyphosis and lumbar lordosis as it can be bent in one plane and preserves the form it is bent into. As a result, it is claimed that it can replicate any curved surface. Additionally, documented reliability for both radiographic and non-radiographic measures of kyphosis, such as flexicurve and marker-based measurements, is often good to excellent (de Oliveira et al., 2012; Grindle et al., 2020).

The finger-to-floor (FTF) test is used to evaluate trunk flexion flexibility; it is a composite motion of the flexibility of the spine, hips, and hamstrings. The FTF test correlated strongly with radiologic measures of trunk flexion. The FTF test is straightforward to administer and has high inter- and intra-rater reliability, validity, and responsiveness (Ekedahl et al., 2012).

Gender disparities have been observed in the sagittal pelvic position, the thoracic curve in the slump sitting position, the lumbar curve in the comfortable standing position, and the slump sitting position. Variations in hamstring flexibility-knee extension and lumbosacral angle throughout greatest trunk forward flexion position were reported in male and female team sports players with low and high hamstring extensibility (Cejudo et al., 2021).

Earlier researches examined the influence of hamstring tightness on lumbar lordosis in cerebral palsy children (McCarthy and Betz, 2000), low back pain symptoms (Allam et al., 2022), lumbar mobility in dentists (Divyashri et al., 2021), and spinal curvatures and pelvic tilting in athletes (Lopez-Minarro and Alacid, 2010; Cejudo et al., 2021). Nevertheless, no prior studies have looked into gender dissimilarities in the association between hamstring tightness and lumbar lordosis, as well as trunk flexibility in young healthy adults. As a result, the goal of this study is to identify any relationships among hamstring tightness, lumbar lordosis, and trunk flexibility in young healthy individuals, as well as to detect differences between males and females.

## Materials and methods

### Participants

One hundred (50 men, 50 women) healthy individuals with hamstring muscle shortening were recruited from Jouf University students to participate in this cross-sectional study. Their ages ranged from 18 to 25 years with a mean ± standard deviation for age is (20.92 ± 1.51) for females and (21.24 ± 1.94) for males and a body mass index (BMI) of (23.05 ± 3.45) for females and (23.41 ± 2.02) for males, with the following inclusion criteria: Healthy individuals with sedentary lifestyle assessed by the International Physical Activity Questionnaire-BREF (IPAQ-BREF), the degree of knee flexion from terminal knee extension (knee flexion angle) was greater than 15° during AKE test and SLR test (Kuilart et al., 2005; Koli and Anap, 2018). Participants with a BMI of 30 or higher, pregnant women, and women who gave birth in the previous year are all excluded, along with other variables that could influence the alignment of the lumbar curve, such as weakness of the abdominal and gluteal muscles (Van Wingerden et al., 2004), and tightness of the hip flexors (Kolber and Fiebert, 2005). The current study was approved by the Qurayyat Health Affairs, Jouf Research Ethics Committee (No: 2023-60). Before administering the tests, each participant signed a written informed consent form, and the instructions, objectives, and steps of the procedure were explained to them.

### Procedures

Participants were divided into two equal groups of 50 each, based on gender: Group A (Female group) and Group B (Male group). Prior to the start of the test, all participants’ demographic data, including height and weight, were recorded. The dominant lower limb of the subject was also determined by having him/her kick a ball in front of him/her. All subjects were asked to warm up on a treadmill for 5 min before the test. Warm-up exercise was in the form of treadmill running at 10 km/h. Participants were instructed to choose a running pace that they were accustomed to while the treadmill speed was gradually raised. Since all of the chosen speeds were within 10% of 10 km/h, this speed was applied to all participants (Young et al., 2004).

1- Hamstring tightness measurement:

The following tests were used to assess hamstring muscle flexibility.

**FIGURE 1:**
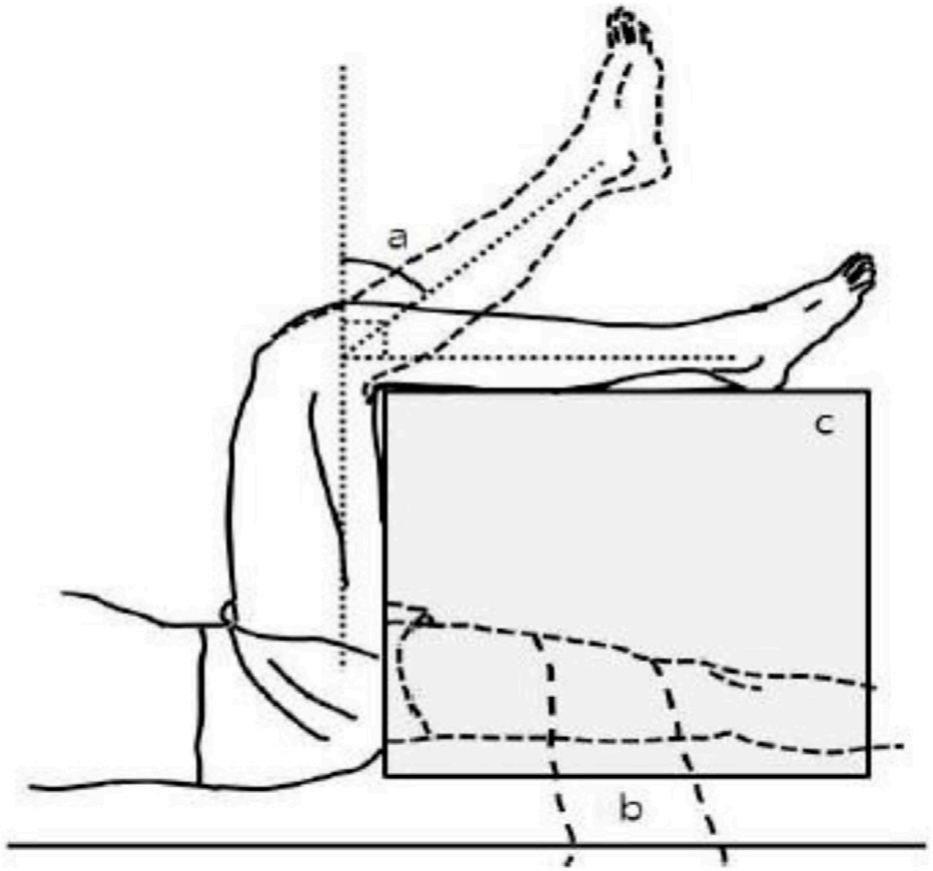
AKE test, **(A)** popliteal angle, **(B)** stabilizing strap, **(C)** adjustable supporting table (Kim et al., 2009).

a) Active Knee Extension Test (AKE) (Figure 1):

The subject is positioned supine on the examination table, without a pillow beneath the head, with the dominant leg’s hip and knee in 90 degrees of flexion. Two Velcro straps held a wooden box to the plinth; a third strap held the participant’s dominant thigh and box; and a fourth strap around the non-dominant thigh to minimize hip flexion during the procedure. For measuring the AKE angle, a standard, double-arm, clear plastic goniometer with a full-circle protractor (1 increments) and (arm length = 31.75 cm) was used. The lateral femoral epicondyle served as the axis of the goniometer with the fixed arm positioned over the thigh and pointed toward the greater trochanter and the moving arm positioned over the leg and pointed toward the lateral malleolus. The subject was then instructed to relax their foot while maximally extending their dominant knee. They held this position for 5 s to allow the AKE angle to be measured (the knee flexion degree from the last knee extension). Each subject completed three trials of the AKE test, each with a 1-min rest period in between, and the mean was recorded for analysis (Hamid et al., 2013).

**FIGURE 2:**
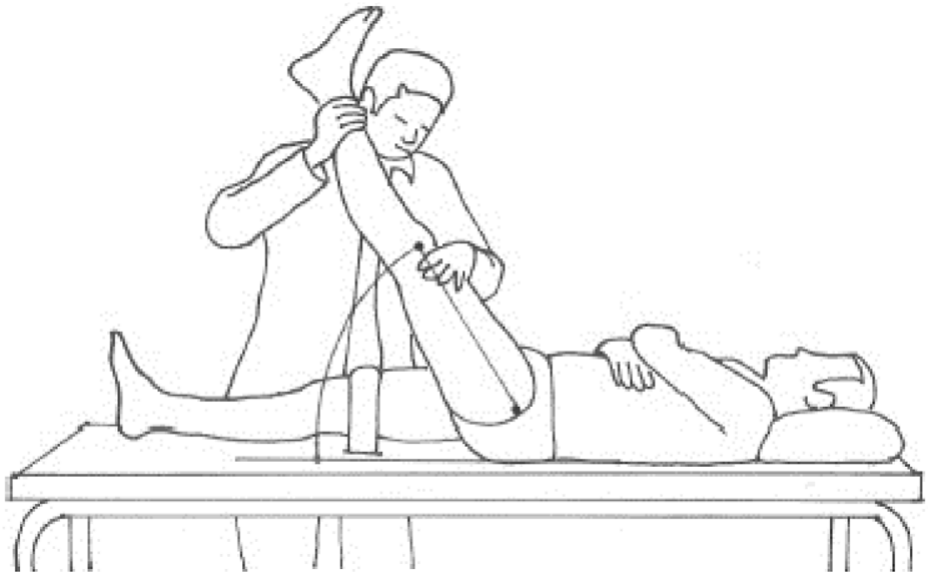
SLR test (de Lucena et al., 2011).

B) Active Straight Leg Raising Test (SLR) (Figure 2):

The subject lay supine on the plinth with a Velcro strap holding the pelvis to the table. Another Velcro strap was used to secure the untested limb in full extension with the hip in the neutral position on the lower third of the thigh. The axis of the universal goniometer is over the greater trochanter of the hip, with the movable arm aligned over the midline of the thigh and pointing toward the lateral femoral condyle, and the fixed arm parallel to the midaxillary line of the trunk. The participant was instructed to relax their foot and actively flex the dominant hip joint while keeping the knee in extension, stopping hip flexion when the knee began to flex or when the participant felt stretch in the hamstrings or lower back, and then holding this position for 5 s to allow the hip flexion angle defect from 90^0^ to be measured (Neto et al., 2015). Each subject completed three trials separated by a 1-min rest period, and the mean was recorded for analysis. The AKE and SLR tests are widely regarded as the gold standards for evaluating hamstring tightness. These assessments’ reliability and validity have been demonstrated in healthy adults (Hansberger et al., 2019).

**FIGURE 3:**
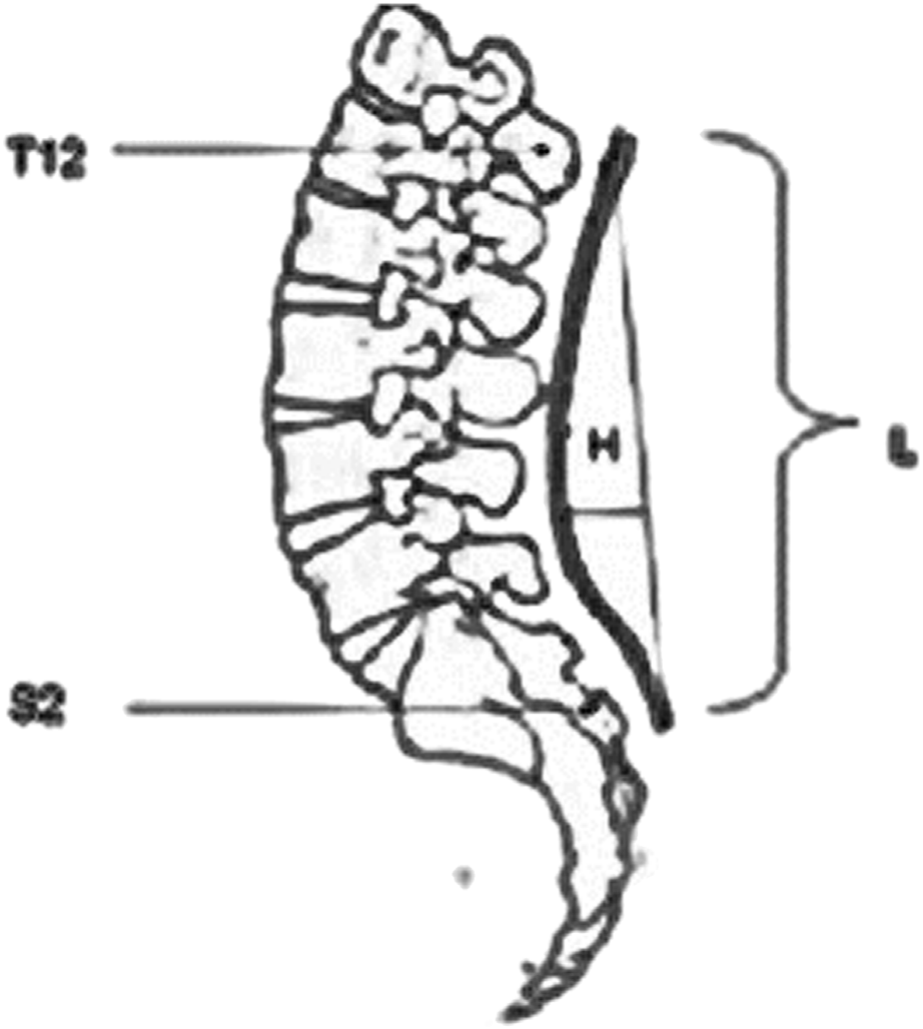
Measurement of lumber lordosis by flexible ruler between T12 and S2 where H is the deepest part of the curve and L is the length of the curve (Rajabi et al., 2008).

2- Lumbar lordosis measurement (Figure 3):

The flexible ruler is an alternative to the radiological Cobb’s method that is non-invasive, cost effective, and accurate. The validity and reliability of the instrument have been established in the thoracic and lumbar regions, and studies have shown a significant link between this technique and Cobb’s angle method (de Oliveira et al., 2012). Each subject stood bare feet, in a neutral upright posture, with body weight distributed evenly on adhesive tape markings on the floor, shoulder width apart between feet. Palpation was used to identify and mark the spinous processes of T12 and S2. The flexible ruler was shaped across the midline of the spine from T12 to S2. The generated curve was traced with a pencil along the flexible ruler on graph paper. The lumbar lordosis angle was measured using the method described by Youdas et al. (2006). To connect the labeled marks of T12 and S2, a vertical line (L) was drawn (total length of curvature). Another perpendicular line (H) was drawn from the curve’s deepest point to the L line. The L and H lines were measured in centimeters. The lumbar lordosis angle (theta) was calculated using the formula: theta = 4 x [arc tan (2H/L)] (Rajabi et al., 2008).

**FIGURE 4:**
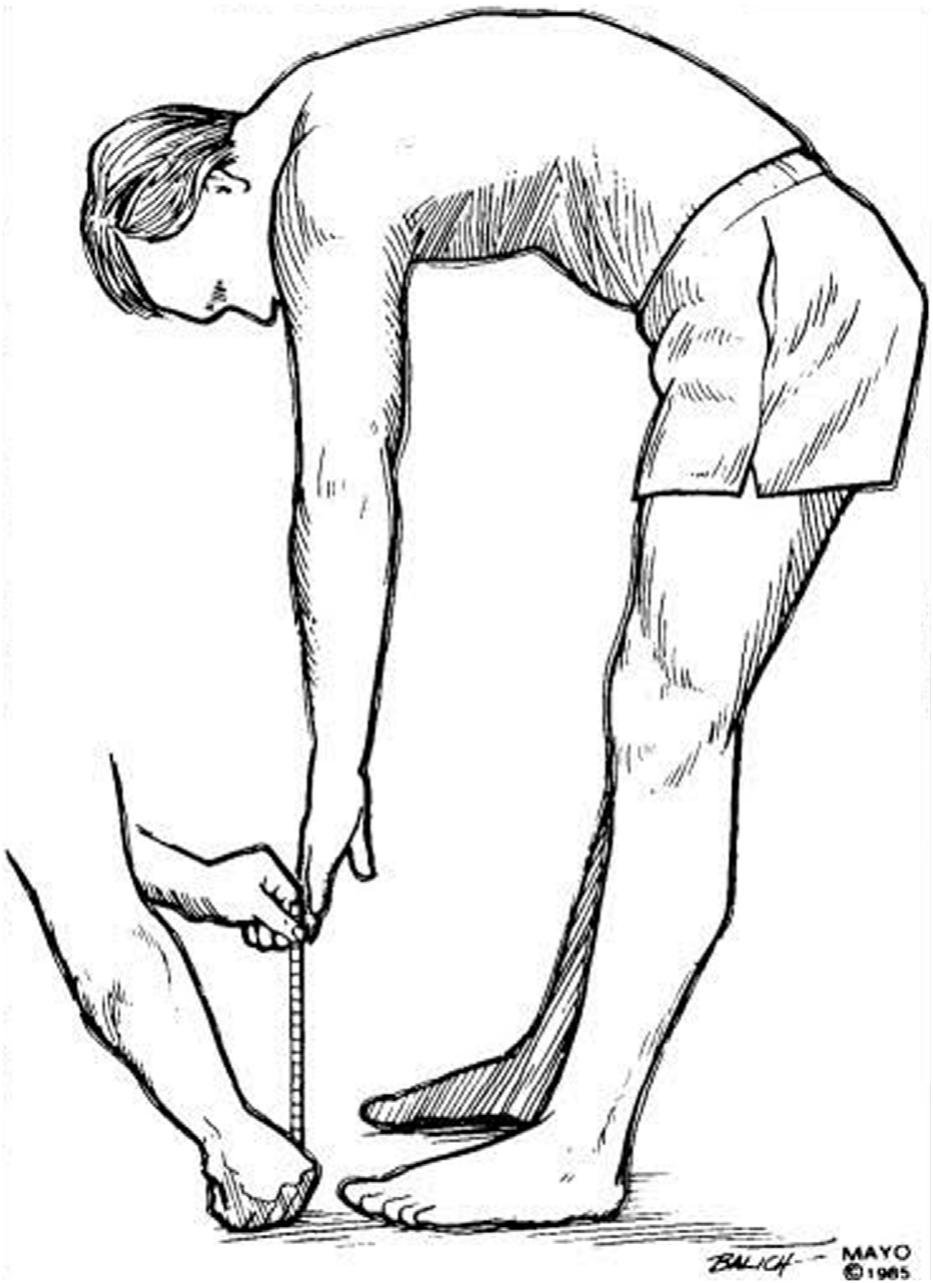
Fingertip to floor test (Merritt et al., 1986).

3- Trunk flexion flexibility measurement (Figure 4):

Fingertip-to-Floor Test (FTF): The participant was instructed to stand barefoot on a small box above the floor, feet together, toes at the box’s edge, bend the trunk forward while keeping the knees extended, place the dominant hand over the other hand, attempting to reach for the toes and stopping when discomfort was felt. A tape measure was used to evaluate the distance in centimeters from the top edge of the box to the tip of the middle finger on the dominant hand. A measurement of “0”revealed that the fingertip was in line with the box’s edge, whereas a positive number revealed that the fingers had not reached the box’s edge. The FTF test is appropriate for clinical practice and therapeutic trials because of its excellent validity, reliability and responsiveness (Perret et al., 2001; Ekedahl et al., 2012).

### Statistical analysis

The unpaired *t*-test was used to compare subject characteristics between groups. The Shapiro-Wilk test was used to ensure that the data was distributed normally. To ensure group homogeneity, Levene’s test for variance homogeneity was performed. The trunk flexion flexibility, Lumbar lordosis angle, SLR, and AKE of females and males were compared using an unpaired *t*-test. The correlation between trunk flexion flexibility, lumbar lordosis angle, SLR, and AKE was determined using the Spearman rank correlation coefficient. The level of significance was set at *p* < 0.05 for all statistical tests. The statistical package for social sciences (SPSS) version 25 for Windows was used to perform all statistical analysis.

## Results

A total of 120 participants were chosen for recruitment based on the inclusion criteria. 100 participants passed the screening and were eligible for the study. Twenty participants were excluded because 7 had hip flexion tightness, 4 had a BMI greater than 30, 4 were pregnant, 3 had given birth in the previous 2 months, and 2 had abdominal muscle weakness. The study procedures were only completed by 100 participants.

### Subject characteristics

Subjects’ characteristics were demonstrated in Table 1. There was no significant difference in age, weight, height, BMI or physical activity between groups A (females) and B (males) (*p* > 0.05).

**TABLE 1:** Basic characteristics of participants.

	Group A (females)	Group B (males)	MD	t- value	*p*-value
	Mean ± SD	Mean ± SD			
Age (years)	20.92 ± 1.51	21.24 ± 1.94	−0.32	−0.91	0.36
Weight (kg)	57.73 ± 8.96	59.19 ± 4.49	−1.46	−1.03	0.31
Height (cm)	158.29 ± 5.08	159.15 ± 4.90	−0.86	−0.86	0.39
BMI (kg/m^2^)	23.05 ± 3.45	23.41 ± 2.02	−0.36	−0.64	0.52
Physical activity (MET)	38.5 ± 112.3	40.3 ± 105.2	−1.8	−0.08	0.93

MET, metabolic equivalent of task; SD, standard deviation; MD, mean difference; *p*-value, probability value.

### Relationship between trunk flexion flexibility and hamstring tightness (SLR and AKE)

There was a moderate positive significant correlation between trunk flexion flexibility (TFF) and both measures of hamstring tightness (SLR, *r* = 0.505, *p* = 0.001; AKE, *r* = 0.549, *p* = 0.001) in group A (females). While, there was no correlation between TFF and both measures of hamstring tightness (SLR, *r* = 0.018, *p* = 0.900; AKE, *r* = 0.053, *p* = 0.717) in group B (males) (Table 2).

**TABLE 2:** Correlation between trunk flexion flexibility and hamstring tightness.

Trunk flexion flexibility (TFF)				
Hamstring tightness	Group A (females)		Group B (males)	
	r value	*p*-value	r value	*p*-value
SLR	0.505	0.001	0.018	0.900
AKE	0.549	0.001	0.053	0.717

r value: Spearman rank correlation coefficient, *p*-value: Probability value Relationship between lumbar lordosis and hamstring tightness (SLR, and AKE).

In group A (females), there was no correlation between lumbar lordosis angle and the SLR measure of hamstring tightness (*r* = −0.189, *p* = 0.190), while there was a no correlation between the AKE measure of hamstring tightness and lumbar lordosis angle (*r* = 0.004, *p* = 0.979). Moreover, there was no correlation between lumbar lordosis angle and both measures of hamstring tightness (SLR, *r* = 0.001, *p* = 0.994; AKE, *r* = 0.047, *p* = 0.747) in group B (males) (Table 3).

**TABLE 3:** Correlation between lumbar lordosis and hamstring tightness.

Lumbar lordosis				
Hamstring tightness	Group A (females)		Group B (males)	
	r value	*p*-value	r value	*p*-value
SLR	−0.189	0.190	0.001	0.994
AKE	0.004	0.979	0.047	0.747

r value: Spearman rank correlation coefficient, *p*-value: Probability value.

### Comparison of both groups’ hamstring tightness (SLR and AKE), lumbar lordosis and trunk flexion flexibility

The unpaired *t*-test revealed that group A (females) had significantly higher hamstring tightness, as the greater degree of SLR and AKE corresponds to greater hamstring tightness, and lumbar lordosis angle than group B (males) (*p* < 0.05). While group A (females) had significantly lower trunk flexion flexibility than group B (males) (*p* < 0.01) (Table 4).

**TABLE 4:** Comparison of hamstring tightness, lumbar lordosis and trunk flexion flexibility between both groups.

	Group A (females)	Group B (males)	MD (95% CI)	t- value	*p*-value
	Mean ± SD	Mean ± SD			
SLR^0^	28.71 ± 8.38	25.45 ± 6.74	3.26 (0.23: 6.27)	2.13	0.030
AKE^0^	37.92 ± 7.21	29.49 ± 5.35	8.43 (5.91: 10.95)	6.64	0.001
Lumbar lordosis^0^	59.00 ± 6.90	52.54 ± 8.27	6.46 (3.43: 9.48)	4.24	0.001
TFF (cm)	6.54 ± 8.89	3.32 ± 3.42	3.22 (0.54: 5.89)	2.39	0.018

SD, standard deviation; MD, mean difference; CI, confidence interval; *p*-value, probability value.

## Discussion

The gender differences found in the majority of the variables measured were the study’s main point. As a result, all variables in this study were analyzed specifically according to gender to determine whether hamstring tightness causes sagittal discrepancy of the lumbar curve and a decrease in trunk flexibility.

The study’s main findings include no correlation between lumbar lordosis angle and SLR as well as AKE test values in females and males. These findings are in line with those of earlier investigations, which found that rowers’ (Stutchfield and Coleman, 2006) and young athletes’ hamstring tightness did not result in lumbar sagittal misalignments in standing positions (Lopez-Minarro and Alacid, 2010; López-Miñarro et al., 2010; Muyor et al., 2012). Lopez-Minarro et al. (2012) revealed no significant differences in hamstring extensibility, thoracic and lumbar flexion in Paddlers standing. Nevertheless, kayakers and canoeists with lower hamstring extensibility had more lumbar flexion and posterior pelvic tilt during training. Other studies indicate low correlation values between hamstring extensibility and lumbar spine position through maximal flexion movements of the trunk with extended knees (Rodríguez-García et al., 2008; López-Miñarro et al., 2009). On the other hand, McCarthy and Betz (2000), observed a link between increasing hamstring tightness and decreasing the angle of lumbar lordosis, as measured by the popliteal angle, in children with cerebral palsy, particularly in the sitting position, as these patients sit with hypo-lordotic or kyphotic lumbar spines. This could be because cerebral palsy is associated with higher degrees of hamstring tightness. Unfortunately, no previous research has been conducted to detect gender differences in the correlation between hamstring tightness and lumbar lordosis in healthy adults.

Additionally, the results of this research indicated that in females, SLR and AKE values showed a moderately positive significant correlation between hamstring tightness and trunk flexibility. While there was no correlation between trunk flexibility and hamstring tightness in males, according to SLR and AKE values. In agreement with our results, Divyashri et al. (2021) encountered a weak positive correlation between dentists’ hamstring tightness and the flexion of their lumbar spine. Female dentists had a higher prevalence of tight hamstrings and a reduction in lumbar spine range of motion than did male dentists. However, there was no statistically significant distinction between the participants who were male and female. The findings point to a significant amount of hamstring and lumbar flexion range of motion tightness by the dentist, which can act as a warning sign for the need for lumbar treatment. Additionally, Stutchfield and Coleman (2006) reported no relationship between hamstring flexibility and lumbar flexion in male rowers. Individuals with greater hamstring flexibility, as in the male group, would have been expected to exhibit less lumbar flexion excursion when completing forward reaching tasks than those with limited hamstring flexibility, as in female group, according to the theory that increased hamstring flexibility decreases the amount of lumbar flexion required during forward reaching tasks (Johnson et al., 2010; Reis and Macedo, 2015). These studies, however, did not compare the outcomes between males and females.

Regarding the differences between male and female, the current study’s findings revealed that females had a greater significant decrease in hamstring and trunk flexibility than males. Moreover, females had a more significant increase in lumbar lordosis angle than males. These findings are consistent with previous research (Divyashri et al., 2021; Pereira et al., 2021). This may be explained by gender differences in anatomy, such as muscle mass percentage which is greater in males, sexual dimorphism of the pelvic architecture, lower limb length, and lower center of gravity, hormonal effects, muscle characteristics, like as muscle stiffness, and basic recruitment patterns such as walking, bending, and reaching. Gender differences in factors related to trunk muscle loads were observed in the spine (Thomas et al., 1998; McPherson et al., 2020). As a result, the differences in hamstring flexibility between men and women may have a different effect on sagittal spinal curves and trunk flexibility. Also, these disparities may be the result of anthropometric differences rather than fundamental differences in muscle characteristics between sexes. These findings show that the male knee flexor musculature may be better suited for injury protection, but that gender differences in knee flexor stiffness are almost fully explained by greater male mass and height (Blackburn et al., 2004). On contrary, other studies found that females had greater flexibility than males as measured by AKE and SLR tests in healthy adults (Youdas et al., 2005) which may be due to the difference in sample size and the wide range of age of participants (20–79 years). The flexibility was found to be higher in female athletes than males (Beynnon et al., 2005; Faherty et al., 2019), which may be because both male and female soccer players frequently suffer from hamstring muscle strains. Previous research has linked changes in hamstring flexibility and range of motion to hamstring muscle strain.

This study’s findings are consistent with other studies that found males to be more flexible than females in flexion of lumber spine in young adults and workers (Chung and Wang, 2009; Moromizato et al., 2016). Females have a shortened spinal column and a larger lumbar lordosis than males, which is thought to account for their lower trunk flexion and rotation. A kinematic analysis of going up from a chair revealed that lumbar spine flexion occurs simultaneously with hip (Fotoohabadi et al., 2010), implying that lumbar spine flexion compensates for hip joint motion inflexibility in males.

In regard to gender disparities in sagittal spinal and spino-pelvic alignment parameters, Vialle et al. (2005), found that female subjects had greater value in lumbar lordosis and pelvic occurrence. Yukawa et al. (2018), also found a significant gender difference in cervical lordosis, lumber lordosis, and pelvic tilt. Hamstring muscle tightness reduces pelvic mobility and the angle of lumber lordosis in the standing position. As a result, the pressure distribution of the spine is inevitably altered biomechanically, resulting in spinal disorders. Poor hamstring extensibility has thus been linked to thoracic kyphosis, spondylolysis, disc herniation, changes in lumbopelvic rhythm, and low back pain. Similarly, people with tight hamstrings have trouble walking, are more prone to falling, and are more likely to sustain musculoskeletal injuries (Erkula et al., 2002; Kendall and McCreary, 2005). Physiotherapists can assist men and women suffering from hamstring tightness by analyzing these factors and preventing complications caused by these changes.

This study has some limitations, including a small sample size and the inclusion of only healthy adults. As a result, we cannot generalize the findings to other people. Other factors that may result from hamstring tightness were not considered in this study, such as pelvic incidence, thoracic kyphosis angle, and low back pain. In addition the authors did not use radiograph method for assessment of lumber lordosis angle to detect if the changes caused by soft tissue or due to radiological differences. Future researches recommended to evaluate gender differences in different age groups and compare individuals with and without low back pain. It is also suggested to investigate other factors to determine their relationship with hamstring tightness and to use radiograph method to detect the gender differences in lumber lordosis and to evaluate the correlation between hamstring tightness and the angle of lumber lordosis.

## Conclusion

There is no association between hamstring tightness and the degree of lumbar lordosis in both males and females. However, there is a significant correlation between trunk flexibility and hamstring tightness in females but not in males. Furthermore, healthy females have less hamstring and trunk flexibility than healthy males. While, females have a greater angle of lumbar lordosis than males. Whereas, the results for lumber lordosis relied on measurement with a flexible ruler, the authors suggest for further studies to measure using radiographs.

## Data Availability

The original contributions presented in the study are included in the article/Supplementary Materials, further inquiries can be directed to the corresponding author.
